# Depth-Dependent Mineral Soil *CO*_2_ Production Processes: Sensitivity to Harvesting-Induced Changes in Soil Climate

**DOI:** 10.1371/journal.pone.0134171

**Published:** 2015-08-11

**Authors:** Lisa Kellman, Amy Myette, Hugo Beltrami

**Affiliations:** 1 Department of Earth Sciences, St. Francis Xavier University, Antigonish, Nova Scotia, Canada; 2 Climate & Atmospheric Sciences Institute, St. Francis Xavier University, Antigonish, Nova Scotia, Canada; 3 Centre pour l’étude et la simulation du climat à l’échelle régionale (ESCER), Université du Québec à Montréal, Montréal, Québec, Canada; University of California Davis, UNITED STATES

## Abstract

Forest harvesting induces a step change in the climatic variables (temperature and moisture), that control carbon dioxide (*CO*_2_) production arising from soil organic matter decomposition within soils. Efforts to examine these vertically complex relationships *in situ* within soil profiles are lacking. In this study we examined how the climatic controls on *CO*_2_ production change within vertically distinct layers of the soil profile in intact and clearcut forest soils of a humid temperate forest system of Atlantic Canada. We measured mineral soil temperature (0, 5, 10, 20, 50 and 100 cm depth) and moisture (0–15 cm and 30–60 cm depth), along with *CO*_2_ surface efflux and subsurface concentrations (0, 2.5, 5, 10, 20, 35, 50, 75 and 100 cm depth) in 1 m deep soil pits at 4 sites represented by two forest-clearcut pairs over a complete annual cycle. We examined relationships between surface efflux at each site, and soil heat, moisture, and mineral soil *CO*_2_ production. Following clearcut harvesting we observed increases in temperature through depth (1–2°C annually; often in excess of 4°C in summer and spring), alongside increases in soil moisture (30%). We observed a systematic breakdown in the expected exponential relationship between *CO*_2_ production and heat with mineral soil depth, consistent with an increase in the role moisture plays in constraining *CO*_2_ production. These findings should be considered in efforts to model and characterize mineral soil organic matter decomposition in harvested forest soils.

## Introduction

Forest soil organic matter (SOM) represents an important global carbon (C) reservoir [1, 2]. There have been increased calls for an improved understanding of the importance of forest soils as sinks (e.g. [3]) and stores of C, and for a more complete evaluation of mineral SOC stocks in establishing policies related to forest management, C accounting, and bioenergy production (e.g. [4]).

Clearcut harvesting is a standard management practice in many temperate forests that generates a step changes in the soil climatic and biological factors that control C transformations, yet our current understanding of how this activity alters soil climate and patterns of decomposition-sourced carbon dioxide (*CO*_2_) through depth in soil profiles remains poorly understood and quantified. Following harvesting, a pulse of C [5, 6] arising from the decomposition and destabilization of harvest residues and SOM in forest soils has been observed [7]. The majority of respired *CO*_2_ from SOM decomposition is derived from a small fast cycling labile pool [8], with the production of *CO*_2_ highest in the surface soil layers and declining with depth (e.g. [9]). Mineral soil horizons can hold a significant proportion of the total SOM [1], and represent pools that differ in their quality [10] and susceptibility to decomposition. Deeper mineral SOM pools have generally been considered unavailable for decomposition through physical separation, or due to inherent chemical recalcitrance [11, 12]. Recent evidence, however, challenges traditional views of SOM stability [13–16], suggesting mineral C stores may be more susceptible to shifts in soil environmental conditions than previously thought. In fact, recent studies have documented mineral SOM profile losses in the decades following clearcut harvesting in temperate forests of north eastern North America [17–19], with isotopic evidence pointing to increased decomposition rates following harvesting [17, 20], particularly within the organo-mineral fraction [21]. Previously, these mineral SOM pools had been assumed to represent a stable fraction that would persist over the timescales of a complete forest harvest cycle. These recent shifts in our understanding of mineral SOM stability suggest a greater potential than previously realized for SOM destabilization, and points to the need to evaluate deep mineral SOM decomposition rates following this disturbance.

The primary role of temperature in controlling SOM decomposition, and the exponential nature of this relationship and its theoretical underpinnings have been well established (e.g. [22–24]). Soil moisture can play a key role in determining temperature-respiration responses in soils (e.g. [25, 26]), as microbial activity can be altered by shifts in water content that affect solute and oxygen diffusion, thereby changing substrate supply and decomposition rates [27, 28]. While soil temperatures are expected to increase in recently harvested sites [29, 30] altering rates of soil respiration, soil hydrological characteristics will change at the same time due to reduced transpiration following vegetation removal [31–33], and may act to either offset or enhance the effects of increased soil temperature on SOM decomposition.

In clearcut soils where the aboveground vegetation has been removed, soil *CO*_2_ efflux arises solely from the decomposition of SOM via heterotrophic respiration of bacteria and fungi. Intact forest soils, however, also release a *CO*_2_ efflux component arising from autotrophic respiration from roots, the release of root exudates, and associated rhizosphere organisms [34–37]. These root-associated processes can play an additional role in SOM decomposition through priming effects [37, 38], and complicate efforts to isolate *in situ* changes in climate-driven SOM decomposition processes. Efforts to separate these components of soil respiration are challenging in the field setting [34, 36, 39].

While soil *CO*_2_ exchange dynamics are most often examined using soil surface efflux measurements, the addition of subsurface *CO*_2_ profile concentration data can yield information about physical controls on these exchanges through depth in soils [23, 40, 41]. A layered mineral soil *CO*_2_ production model can allow depth-specific relationships to be developed between *CO*_2_ production rates and the physical environment [40, 42]. If scaled to soil efflux measurements, problems associated with modeled diffusivity estimates [43] can be minimized, and further insight into climate-driven processes within soil profiles may be provided. For example, if soil thermal properties exerted a dominant control on *CO*_2_ production, an exponential relationship might be expected to provide the best predictive model [22]. If other environmental factors were dominating the *CO*_2_ production dynamics, we might observe a breakdown in the exponential relationship. Although vertical concentration data can be spatially variable, common patterns are generally evident through depth and across independent plots within sites [44]. Thus, developing hypotheses about how environmental factors ultimately control soil *CO*_2_ production processes in discrete subsurface soil layers can be carried out using plots where detailed observations of gas concentrations and corresponding soil climate data are available.

The objective of this study is to quantify changes is relationships between soil climate (temperature and moisture) and *CO*_2_ production within vertically distinct layers of the soil profile following clearcut harvesting in a humid temperate forest system. We hypothesize that increases in soil heat and moisture in soils following clearcut harvesting will alter the quantitative relationships between these variables and soil *CO*_2_ production within vertical soil layers in a manner that reflects the increasing constraint played by soil moisture through depth and following clearcut harvesting. In order to accomplish these objectives we measure soil surface *CO*_2_ efflux and use 1 m deep soil pits at 4 sites represented by two forest—clearcut pairs instrumented to monitor soil temperature, moisture and *CO*_2_ concentrations over a complete annual cycle in the Acadian Forest Region of Atlantic Canada. The Acadian Forest forest represents a range of mixed forests, many dominated by red spruce. The red spruce forests of this region have been the subject of several other soil C studies (e.g. [17, 18, 20, 21]) as they provide useful model systems for understanding SOC dynamics in moist temperate forest soils subjected to routine clearcut harvesting disturbances [45].

## 1 Methods

### 1.1 Study Site

The study was conducted between August 2003 and August 2004 at two recent (1 y) and late (> 50 y) post harvest successional forest pairs typical of the Acadian Forest Region in Northeastern Nova Scotia, Canada. The paired forest- clearcut sites, Lakevale (45°45′′6′′N, 61°56′46′′W) and Pomquet (45°39′′22′′N, 61°50′32′′W) are located less than 20 km distance apart, while at each site the forest-clear cut pair is separated by approximately 200 m and 5 km for Lakevale and Pomquet respectively. The study was carried out on private land with permission of the land owners. Both sites are in a coastal region and close to sea level. Soils of both sites are classified as podzols under the Canadian System of Soil Classification. The Lakevale paired site soils (LF—intact forest; LCC—clear cut forest), are Millbrook soils with brown loam over reddish brown gravely clay loam formed on a parent material of brown shales and sandstone [46]. The Pomquet paired site soils (PF—intact forest; PCC—clear cut forest) are Queens soils with light brown clay loam over reddish brown clay loam formed on a parent material of dark reddish brown clay loam till derived from brown shale [46]. The finer textured Pomquet soils are more poorly drained than the sandy Lakevale soils (for additional soil profile textural information refer to [44]). The depth to the organo-mineral intereface (herein referred to as the 0 cm mineral soil depth) from the land surface averaged 6.5 cm and 5.5 cm for forest and clear cut sites respectively. Both paired sites receive mean annual precipitation of approximately 1290 mm and have mean annual surface air temperatures of 5.5°C.

The forest at LF is approximately 85-year old and consists of balsam fir (*Abies balsamea (L.)Mill*, 38%), red spruce (*Picea rubens Sarg*., 35%) and white spruce (*Picea glauca Moench Voss*, 11%). The LCC site was clear cut in the spring of 2002 and sprayed with a herbicide (Vision glyphosate (N-phosphonomethyl glycine), Monsanto Corp., St. Louis, MO) in late summer 2003 to hinder growth of deciduous plants. The new growth consists of a mixture of raspberry (*Rubus idaeus L*.), red maple (*Acer rubrum L*.), and trembling aspen (*Populus tremuloides Michx*.). The vegetation at PF is approximately 55 yr old, and consists of mainly red spruce (86%). Other plants at PF include trembling aspen (5%), sugar maple (*Acer saccharum Marsh*, 4%) and paper birch (*Betula papyrifera Marsh*, 4%). The PCC site was clear cut in the spring of 2002 and was beginning to regenerate with ash (*Froxinus*) and spruce (*Picea*) seedlings. No herbicide was applied to this site.

### 1.2 Meteorological Stations

Each site (LF, LCC, PF and PCC) is equipped with a meteorological station monitoring standard aboveground climate information and detailed subsurface thermal and moisture regimes [47, 48]. The instrumentation consists of a control unit and a solar panel, two Cambell Scientific (CS) 107 air temperature probes at a height of 2 m enclosed in radiation shields, and six CS 107b soil temperature probes at depths of 0, 5, 10, 20, 50 and 100 cm. The stations are operated by CS CR-10 data-loggers powered by rechargeable batteries and solar panels. Instruments are sampled every 30 seconds and five minutes averages of all sensors are recorded. The accuracy of the CS107 air temperature probe and CS107b soil temperature probes is < ±0.2 K. Most of this error corresponds to the offset from the interchange of the probes, but with a single point calibration, it is possible to eliminate the probe offset and the working accuracy is reduced to better than < ±0.1 K. The temperature probes were inserted horizontally into the vertical wall of a soil pit. Soil moisture, measured using time domain reflectometry (TRD) probes 30 cm in length were installed at depths of 0 cm (organic mineral interface) and 30 cm deep, at approximately a 45 and 0 degree angle to vertical, respectively. This provided a shallow (0–15 cm) and deep (30–60 cm) soil moisture estimate at each site. Volumetric soil moisture (acquired from saturated soil volumetric moisture contents) was converted to percent water filled pore space (%WFPS) based upon total pore space estimates for the purpose of quantifying the relationships between moisture, temperature and surface flux between sites. Percent air filled pore space (%AFPS) represents the difference between total pore space and WFPS.

### 1.3 Gas Sampling

Subsurface soil air *CO*_2_ concentrations were measured at 0, 2.5, 5, 10, 20, 35, 50, 75 and 100 cm below the organic-mineral interface in each soil pit using individual 50 cm long polyvinylchloride (PVC) samplers (internal volume of 56.5 cm^3^) installed horizontally into the undisturbed wall of a soil pit. A long narrow perforation in the PVC tube that was covered by a breathable water resistant porous membrane allowed soil air to diffuse into the sampler. Microbore tubing connected the sampler to the surface where they were fitted with 3 way valves in order to purge the length of microbore tubing prior to gas sample extraction and to ensure no exchange with atmospheric air. The pits were excavated carefully, the holes for the samplers drilled into the side of the pit, and the samplers fully inserted laterally into the undisturbed soil profile adjacent to the soil pits. This was done to minimize any potential disruption to *CO*_2_ concentration profiles caused by the excavation and backfilling of the adjacent soil pits. The microbore tubing and the valves were housed at the surface in a water tight enclosure. Gas samples were collected in N_2_ purged and evacuated 6ml Exetainer vials (Labco, UK). Triplicate samplers were installed at 2.5 cm, 10 cm and 50 cm depth within each pit of each site in order to estimate variability of *CO*_2_ concentrations within single soil pits.

Surface flux measurements (n = 10 per site per sampling day randomly located within 10 meters of the soil pit) were obtained from all 4 sites using non-steady state vented surface flux chambers [42], constructed of PVC tubing (volume of 0.00109 m^3^; surface area of 0.00754 m^2^). Samples from the chamber headspace were collected in 6 mL N_2_ purged and evacuated Exetainer vials.

Gas sampling was conducted weekly during the growing season and approximately every two weeks during the winter months between August 2003 and August 2004. Paired sites were sampled within less than an hour of each other at the same day on each sampling date, alternating between mid-morning and mid-afternoon at each set of paired sites. All gas samples were returned to the laboratory and analyzed on a Licor LI-7000, *CO*_2_/H_2_O infrared gas analyzer in continuous flow mode (carrier gas N_2_; sample volume 1 ml) within seventy-two hours of collection. Errors associated with gas sample collection, handling and analysis were less than 10%. Cross calibrations with a Licor LI-8100 automated surface flux system was made and a correction applied for observed underestimations in surface flux measurements in manual chambers.

### 1.4 Soil Thermal Regime

Temperature data from each site were used to estimate the net annual and seasonal mean temperatures, and the differences between paired sites used to quantify changes in the temporal patterns in the ground thermal regime. Soil temperature is generally used to express changes in the thermal regime of soils due to disturbance [49, 50] however, because of the high frequency variability in soil temperature, it is often desirable to consider a quantity with a well-defined physical meaning which integrates thermal variability over a time period and depth interval, and provides a robust index of the thermal state of the soil profile. Soil profile heat content and its variation have been previously found useful to discern relationships between soil *CO*_2_ dynamics and the thermal regime of the soil [40, 42]. Variation of the soil profile heat content to a depth of 1 meter (or heat anomalies) are, for typical soil properties, a representation of the mean thermal regime of the subsurface for the previous day. In fact, integrating over the soil profile to determine the heat, is a physically meaningful way to filter temperature data by preserving the long-term trend in a time scale of days. The subsurface heat content, *Q*_*s*_, is determined by (e.g. [51, 52]):

$$\begin{array}{c} Q_{s}=\rho C_{0}\int_{0}^{z_{max}}T\left(z\right)dz, \end{array}$$ (1)

which for the discrete sampling array in this study *Q*_*s*_ can be written as:

$$\begin{array}{c} Q_{s}=\rho C_{0}\sum_{i=1}^{n}T_{i}\frac{\left(z_{i+1}-z_{i}\right)+\left(z_{i}-z_{i-1}\right)}{2}, \end{array}$$ (2)

where, *Q*_*s*_ is in *Jm* ^−2^, *n* is the number of sample levels, *ρ* is the density and *C*_0_ is the specific heat; *ρC*_0_ is the volumetric heat capacity of the soil in *Jm* ^−3^ *K* ^−1^, *T* is the temperature in *K* and *z* is the depth (*m*) below ground surface. The heat anomalies were determined as the difference between the annual mean of absolute heat and daily absolute heat measurements at each site.

### 1.5 Soil *CO*_2_ Dynamics

Soil carbon dioxide dynamics were examined using mean *CO*_2_ surface flux estimates and subsurface *CO*_2_ soil gas profile production data. In order to capture the general trends in subsurface *CO*_2_ concentration and reduce the effects of instrument malfunction and outliers in the dataset, subsurface concentration profiles from each sampling date and site within the mineral soil were smoothed with a robust statistical fitting technique that uses an iteratively reweighed least squares algorithm, with the weights at each iteration calculated by applying the Tukey’s biweight (bisquare) function to the residuals from the previous iteration [53]. The results are less sensitive to outliers in the data when compared with ordinary least squares regression.

Carbon dioxide production at each depth is calculated as the difference between the flux across soil layers, in other words, the output flux from layer i into the overlying layer i +1 (Fi) minus the input into layer i from the underlying layer i 1 (Fi-1) from the surface to maximum sampling depth,

$$\begin{array}{c} p_{CO_{2}}=F_{i}-F_{i-1}, \end{array}$$ (3)

where p_*CO*_2__ is production of *CO*_2_, F is *CO*_2_ flux density (g m^−2^ s^−1)^ and i represents a certain soil layer at depth z.

For a specific soil layer, *i*, the flux, *F*_*i*_, is determined from Fick’s Law in one dimension:

$$\begin{array}{c} F_{i}=-D\frac{\partial C}{\partial z}, \end{array}$$ (4)

where D is the diffusivity (m^2^ s^−1^), C is the *CO*_2_ concentration (*gm* ^−3^) and z is depth (m).

Combination of (3) and (4) [54] yields:

$$\begin{array}{c} p_{CO_{2i}}=\left[D_{e_{i}}\left(\frac{C_{i}-C_{i-1}}{\Delta z}\right)\right]-\left[D_{e_{i+1}}\left(\frac{C_{i+1}-C_{i}}{\Delta z}\right)\right], \end{array}$$ (5)

where *C*_*i*_ and *D*_*e*_*i*__ are the concentration and effective diffusivity for layer i, respectively. As in [40, 42], production was assumed ≥ 0.

Effective diffusivity is calculated using a modified Millington relationship [55] that includes an expression for aqueous diffusion,

$$\begin{array}{c} D_{e}=\frac{\frac{\theta_{w}^{\frac{10}{3}}D_{fw}}{H}+D_{fg}\theta_{g}^{\frac{10}{3}}}{\theta_{T}^{2}}, \end{array}$$ (6)

where *D*_*fg*_ is the diffusion coefficient in free air, *D*_*fw*_ is the diffusion coefficient in free water, *θ*_*T*_, *θ*_*w*_ and *θ*_*g*_ are the total, water-filled and gas-filled volumetric soil porosity values respectively, and *H* is the dimensionless form of Henry’s solubility constant for *CO*_2_ in water [56].

Layered mineral soil *CO*_2_ production values generated using the model outlined above were used to estimate proportions of total mineral soil *CO*_2_ production contributing to surface efflux on a given sampling date within the mineral soil. We assumed at all sites that approximately 50% of microbial respiration was generated from the mineral soil, and at forested sites that 50% of surface flux was generated from root respiration annually [34, 57]. These assumptions represent approximations only, and had no bearing upon the weighting of *CO*_2_ production for specific depth intervals, only the absolute values. By doing this we generated *CO*_2_ production values that were constrained by rates of observed soil *CO*_2_ efflux, and which could therefore be used to explore quantitative relationships with climate variables.

Relationships between soil physical parameters (i.e. moisture, temperature and heat anomaly) and *CO*_2_ gas dynamics (i.e. surface flux and vertical production trends) were examined with Sigmaplot and SPSS (SPSS Inc., Chicago, Illinois, USA).

## 2 Results

### 2.1 Changes in soil temperature, heat and moisture due to clearcut harvesting

**Fig 1:**
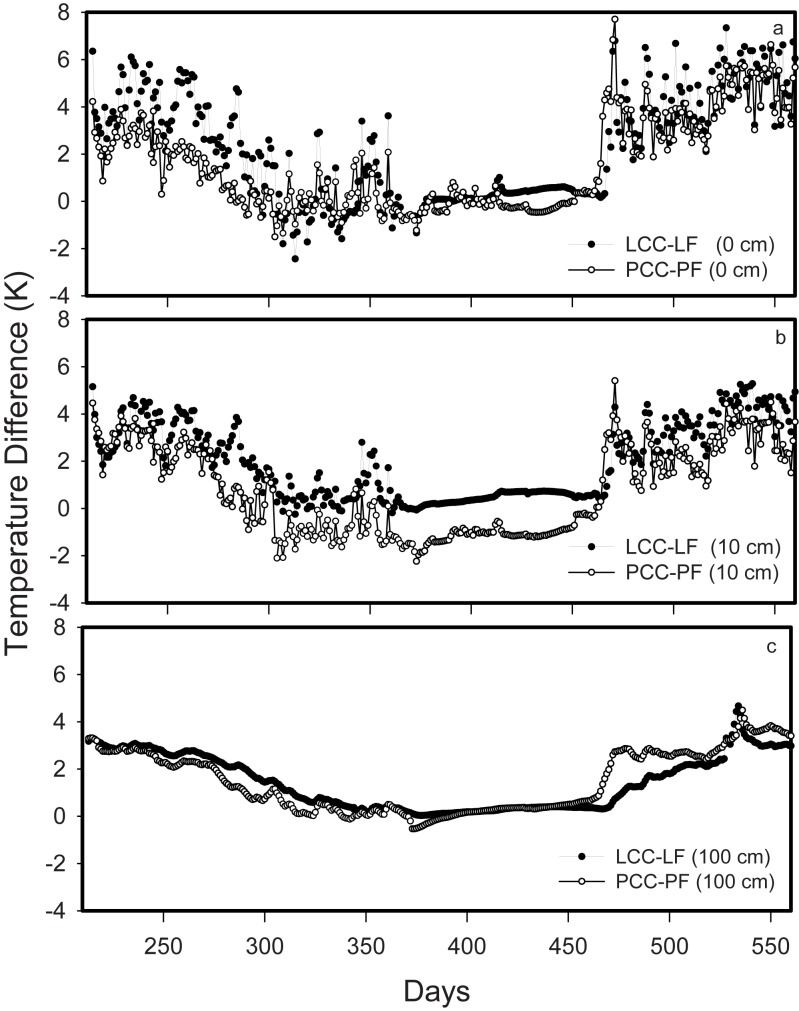
Annual soil temperature difference time series between Lakevale (L) and Pomquet (P) clearcut sites (LCC; PCC) and their forest pairs (LF; PF) for a) 0 cm, b) 10 cm and C) 100 cm depths. The x-axis represents days since Jan 1 of the first year of sampling.

**Fig 2:**
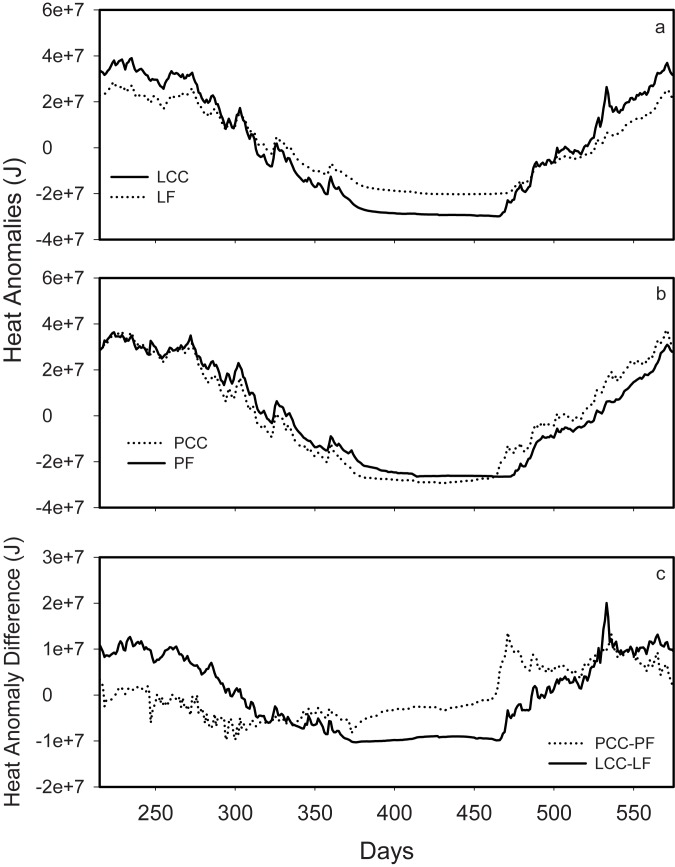
Annual time series of soil profile heat anomalies (J) for a) Lakevale clearcut (LCC) and forest (LF) sites, b) Pomquet clearcut (PCC) and forest (PF) sites, and c) soil profile heat anomaly differences for Lakevale and Pomquet sites. The x-axis represents days since Jan 1 of the first year of sampling.

Measured differences in mean annual soil temperatures between intact forest—clearcut pairs for each sampling depth (0, 10, 100 cm) are on the order of 1.5–2°C (Table 1). Through the study period these differences are not constant (Fig 1), with the greatest soil temperature differences in the upper soil profile often in excess of 6°C during the warmest periods. The calculated soil heat anomalies (Fig 2), demonstrate the greater range in soil heat associated with clear-cutting of the Lakevale sites, however the same ranges are not observed at the Pomquet sites.

**Table 1:** Soil temperature ranges (°C) and annual means through depth at each study site.

	LF			LCC			PF			PCC		
Depth (cm)	Max	Min	Avg	Max	Min	Avg	Max	Min	Avg	Max	Min	Avg
0	20.2	-1.4	6.8	31.7	-1.64	8.9	19.9	-2.21	6.0	28.4	-2.81	8.5
5	19.8	-0.57	6.9	27.8	-0.52	8.9	19.3	-1.04	7.1	26.2	-1.97	8.5
10	19.3	-0.17	6.9	24.6	0.09	8.9	19.3	0.3	7.8	25.2	-0.8	8.7
20	17.3	0.49	6.9	21.2	1.0	8.8	16.8	-0.23	6.6	23.1	0.09	8.5
50	14.4	1.4	6.9	20.7	1.7	8.6	15.2	1.0	6.9	17.8	1.0	8.5
100	12.4	2.2	6.9	15.4	2.6	8.4	13.0	1.6	6.9	15.9	1.6	8.5

**Fig 3:**
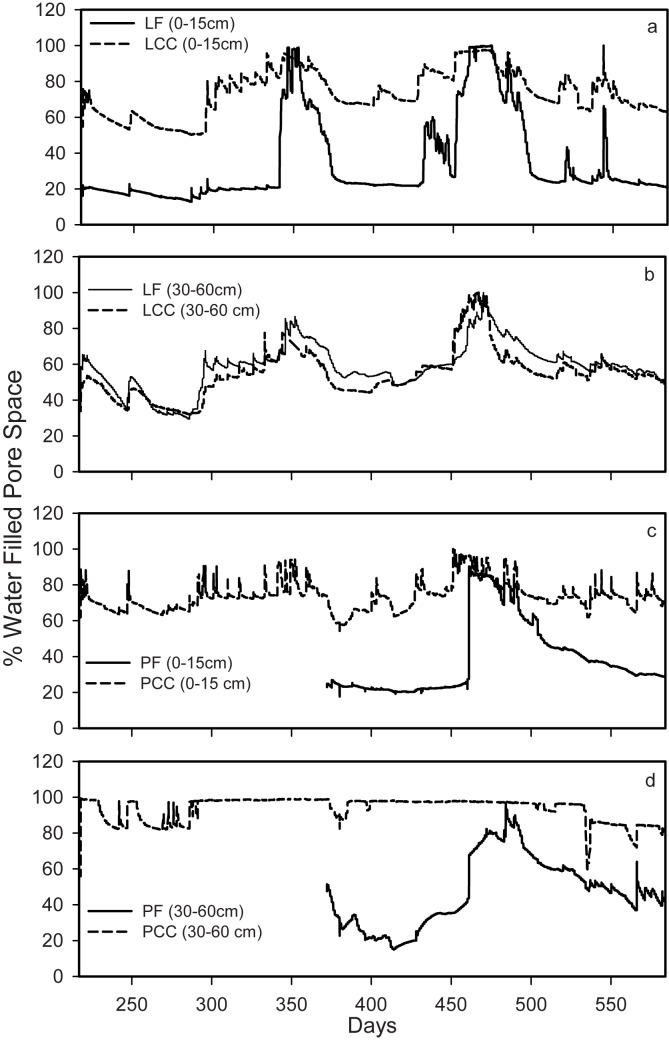
Annual patterns of percent water filled pore space (WFPS) for a) shallow (0–15 cm) Lakevale sites (LCC; LF), b) deep (30–60 cm) Lakevale sites (LCC; LF), c) shallow (0–15 cm) Pomquet sites (PCC; PF), and d) deep (30–60 cm) Pomquet (PCC; PF) sites. The x-axis represents days since Jan 1 of the first year of sampling.

Differences in soil pore space occupied by water (WFPS) at two depths in the soil profile at each site (Fig 3) show overall patterns of increased soil water storage occurring at these sites as a consequence of clearcut harvesting. Over the measurement period, these differences account for an average increase of over 30% WFPS at clearcut sites with the exception of the deep Lakevale sites.

### 2.2 Soil *CO*_2_ Patterns

**Fig 4:**
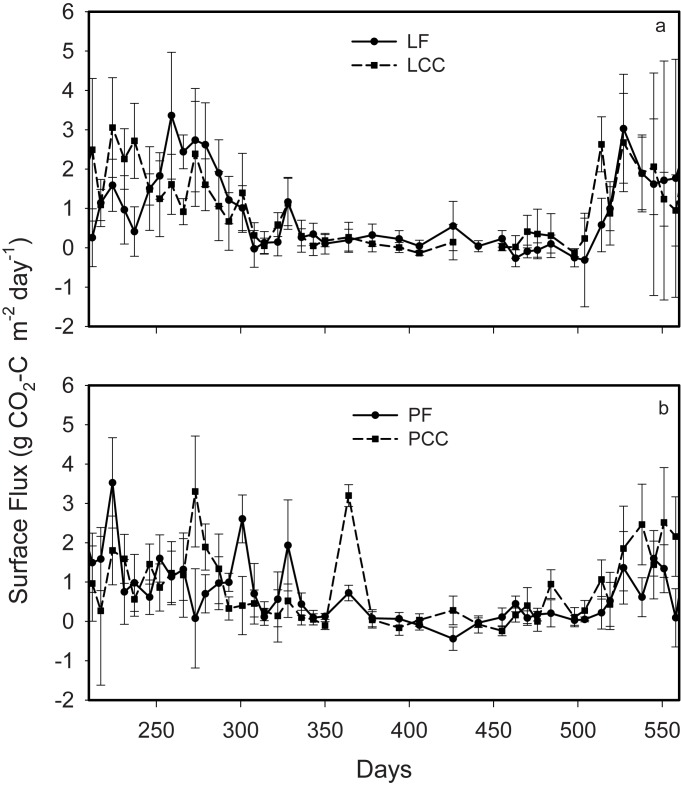
Annual surface *CO*_2_ efflux observations for a) Lakevale (LCC; LF) and b) Pomquet (PCC; PF) sites. The x-axis represents days since Jan 1 of the first year of sampling.

Surface flux patterns for the sites show that all sites are typically sources of *CO*_2_, and that there exists a high within site variability on a given sampling date (Fig 4). Lakevale sites were greater net annual sources of total *CO*_2_ (annual averages of 416 and 342 gC m^2^d^−1^ for LF and LCC respectively) than the Pomquet sites (227 and 287 gC m^2^d^−1^ for PF and PCC respectively).

**Fig 5:**
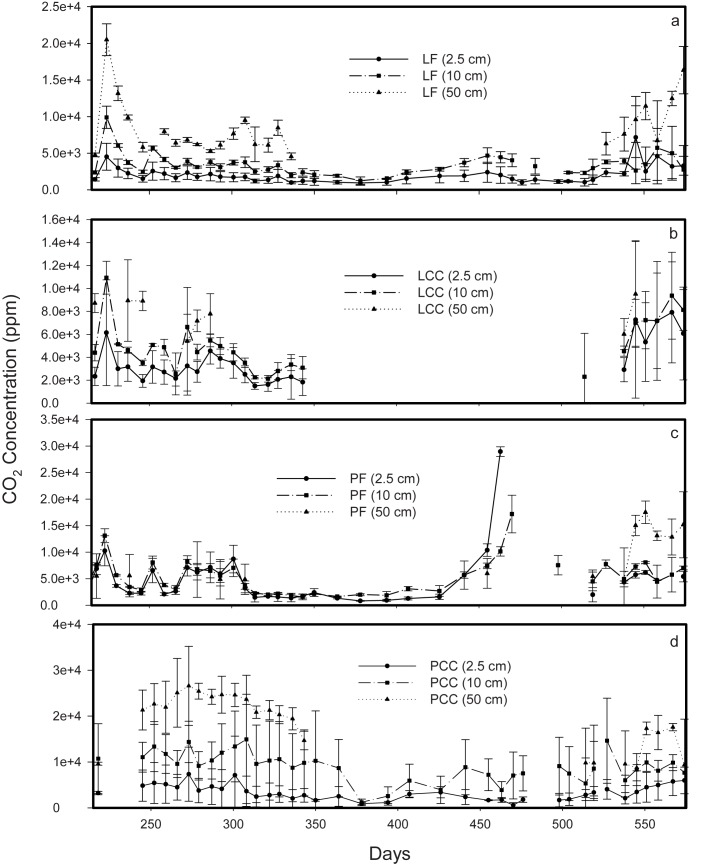
Mean subsurface soil *CO*_2_ concentrations (ppmv) at 2.5 cm, 10 cm and 50 cm depth in the mineral soil for a) Lakevale forest (LF), b) Lakevale clearcut (LCC), c) Pomquet forest (PF) and d), Pomquet clearcut (PCC) sites over the measurement period. The x-axis represents days since Jan 1 of the first year of sampling.

Soil subsurface *CO*_2_ concentrations typically increase through depth in soil profiles (Fig 5). Measurements of profile concentrations from the triplicate samplers at a subset of depths at the sites also point to the high level of variability within a single soil pit (Fig 5; Table 2). For mineral soil depth intervals covering 2.5 cm, 10 cm and 50 cm, the coefficient of variation ranged between 0.16 and 0.62 (Table 2).

**Table 2:** Means, standard deviation (SD) and coefficient of variation (CV) for triplicate *CO*_2_ concentrations over the study period for each site.

*CO*_2_ concentrations (ppmv)									
	2.5 cm			10 cm			50 cm		
	Mean	SD	CV	Mean	SD	CV	Mean	SD	CV
LF	2029	857	0.38	3475	709	0.21	8581	1291	0.16
LCC	3643	1946	0.47	5003	1184	0.26	7812	2325	0.32
PF	4713	940	0.24	5561	880	0.17	11110	3345	0.42
PCC	3370	2252	0.62	9291	5323	0.56	18721	3814	0.25

**Fig 6:**
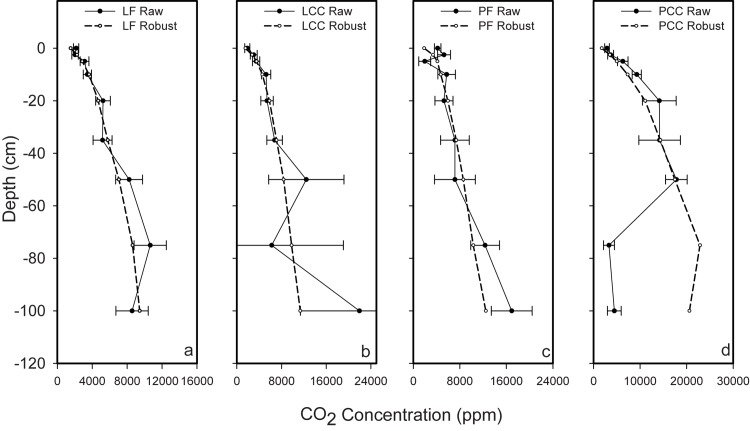
Average annual *CO*_2_ soil air concentration profiles for 0 to 100 cm within the mineral soil for a) Lakevale forest (LF), b) Lakevale clearcut (LCC), c) Pomquet forest (PF), and d) Pomquet clearcut (PCC) sites. Measured mean values (solid line)and robust fit values (broken line) are shown from single pits at each site.

Site-specific averaged annual subsurface concentration profiles (Fig 6) generally show a strong positive gradient, typical of what was observed on individual sampling dates. The exception was PCC where for the majority of the sampling period the water table was close to 50 cm (see Fig 3d). On the few occasions when a sample was obtained below 50 cm depth, concentrations were low. Smoothing of the profiles (dashed lines) allowed the dominant subsurface concentration patterns to be estimated within the mineral soil of all sites. This provided a reasonable approximation of the observed profile patterns, with the exception of PCC below 50 cm depth; therefore *CO*_2_ production estimates at this site were a function of soil *CO*_2_ production above 50 cm depth.

**Fig 7:**
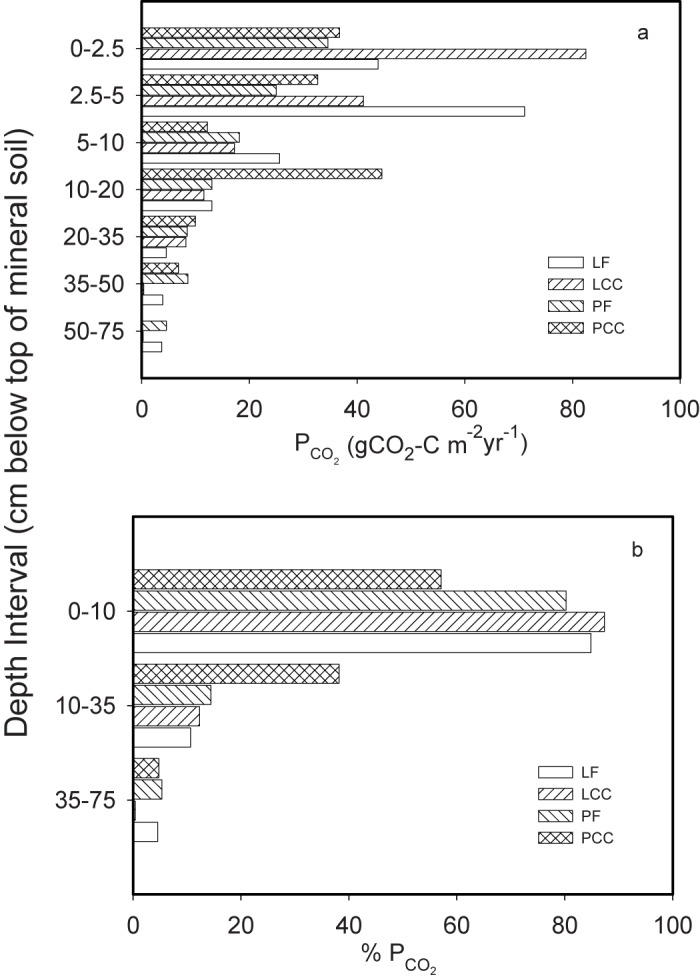
Annual mineral soil *CO*_2_ production estimates for Lakevale (LCC; LF) and Pomquet (PCC; PF) sites for a) all sampled mineral soil depth intervals, and b), as a percent of the total mineral soil profile total for three grouped depth intervals (0–10cm, 10–35cm, and 35–75cm).

Annual summed estimates of vertical soil *CO*_2_ production from each mineral soil layer (Fig 7a), show the dominance of surface processes (and the larger decomposition-sourced *CO*_2_ from the clearcut sites relative to their forest pairs). Proportions from upper, mid, and lower mineral soil profiles (Fig 7b) showed some variability, but highlight the more presistant deep mineral soil source at the forest sites. At the PCC site, this appears to arise from the inhibition of *CO*_2_ production rates due to more frequent saturated soil conditions in the deeper soil profile.

### 2.3 Quantitative relationships between soil climate and *CO*_2_

The relationship between surface efflux and heat anomaly, described using a linear multivariate regression with heat anomaly and soil moisture content as the predictive variables (Table 3), indicate that *CO*_2_ surface flux was primarily driven by soil heat, and that the inclusion of soil moisture did not improve the heat-*CO*_2_ surface flux relationships. An exponential relationship between surface flux and soil heat did not generally improve the relationships ($R_{LF}^{2}=0.50;R_{LCC}^{2}=0.58;R_{PF}^{2}=0.44;R_{PCC}^{2}=0.37;P<0.0001$).

**Table 3:** Results from multivariate regression of *CO*_2_ surface flux as a function of soil heat content and soil moisture for each study site. The relationship is represented by: *SF* = *M*_1_ *Q*+*M*_2_Θ+*B*+*C*, where Q is heat anomaly, Θ is volumetric water content and *M*_1_, *M*_2_, *B* and *C* are constants.

Site	*M*_1_(10^−8^)	*M*_2_	*B*	*C*	*R* ^2^	*P*
LF	4.88	-	0.870	-	0.545	< 0.0001
LCC	3.60	-	0.924	-	0.574	< 0.0001
PF	2.86	-	0.688	-	0.437	< 0.0001
PCC	2.84	-	0.817	-	0.390	< 0.0001

Both exponential and linear models were used to examine relationships between soil heat and *CO*_2_ production within upper (0–10cm), mid (10–35cm) and lower (35–75cm) mineral soil layers at all sites (Table 4). A consistent pattern is evident, with the shallow segments of the soil profile best described by exponential relationships, and deeper profile a linear relationship, or no relationship once the exponential relationship breaks down. The breakdown of the exponential model occurs at a shallower depth interval at clearcut sites than forested sites. The reduced sensitivity to heat through depth in the soil profile, is most evident at the clearcut sites.

**Table 4:** Results of the linear and non-linear soil heat versus soil *CO*_2_ production multivariate regressions for three depth intervals (0–10cm; 10–35cm; 35–75cm) at all sites, forested sites, clearcut sites, and individual sites.

		Heat vs *PCO*_2_				Heat vs *ln*[*PCO*_2_]			
		*PCO*_2_ = *mQ*+*b*				*lnPCO*_2_ = *mQ*+*b*			
Site	Δ*Z*(*cm*)	P-value	R2	m	b	P-value	R2	m	b
All	0–10					< 0.0001	0.438	5.37 ⋅ 10^−8^	-2.006
	10–35	-	-	-	-	< 0.0001	0.144	3.74 ⋅ 10^−8^	-3.570
	35–75	0.001	0.067	5.14 ⋅ 10^−10^	0.015	-	-	-	-
Forest	0–10	-	-	-	-	< 0.0001	0.333	5.58 ⋅ 10^−8^	-2.005
	10–35	-	-	-	-	< 0.0001	0.235	5.02 ⋅ 10^−8^	-3.951
	35–75	0.004	0.088	7.83 ⋅ 10^−10^	0.019	-	-	-	-
Clearcut	0–10	-	-	-	-	< 0.0001	0.581	5.24 ⋅ 10^−8^	-2.010
	10–35	0.001	0.135	3.21 ⋅ 10^−9^	0.103	-	-	-	-
	35–75	0.047	0.048	3.32 ⋅ 10^−10^	0.011	-	-	-	-
LF	0–10	-	-	-	-	< 0.0001	0.539	6.12 ⋅ 10^−8^	-1.590
	10–35	-	-	-	-	< 0.0001	0.407	5.76 ⋅ 10^−8^	-3.760
	35–75	-	-	-	-	-	-	-	-
LCC	0–10	-	-	-	-	< 0.0001	0.71	5.88 ⋅ 10^−8^	-1.814
	10–35	-	-	-	-	-	-	-	-
	35–75	-	-	-	-	-	-	-	-
PF	0–10	-	-	-	-	0.001	0.271	5.44 ⋅ 10^−8^	-2.442
	10–35	0.003	0.206	1.39 ⋅ 10^−9^	0.047	-	-	-	-
	35–75	0.022	0.128	7.52 ⋅ 10^−10^	0.019	-	-	-	-
PCC	0–10	-	-	-	-	< 0.0001	0.475	4.49 ⋅ 10^−8^	-2.198
	10–35	0.003	0.21	5.00 ⋅ 10^−9^	0.155	-	-	-	-
	35–75	0.037	0.107	6.81 ⋅ 10^−10^	0.02	-	-	-	-

## 3 Discussion

### 3.1 Changes in soil climate following clearcut harvesting

As expected, the removal of the vegetation cover following clearcut harvesting produces a significant change in soil thermal and hydrological characteristics (Table 1; Figs 1, 2 and 3). The observations of changes to the soil thermal regime made in this study are consistent with those of other studies investigating changes in soil temperature following forest harvesting [29, 30]. Similarly, it is expected that removal of the forest vegetation, in addition to warming the ground, will result in reduced transpiration within the rooting zone of soils, thus altering the hydrological system in these soils [31–33]. While over the measurement period the soil moisture differences account for an average increase of over 30% WFPS at these clearcut sites, the exception is the deep LCC site, which responded similarly at depth. The soils of this region are moist, typical of a region dominated by humid temperate forests, so increased soil moisture following clearcut harvesting is not unexpected. These sites illustrate responses that are consistent with the textural differences of the soils; specifically, the coarser textured LCC site would be expected to drain more rapidly than PCC soils in response to a proportional increase in soil water inputs. The observed patterns suggests that the hydrological component of the soil climatic response to the harvesting disturbance is more prone to site-specific characteristics that control water transport dynamics within the vadose and shallow groundwater zone. In contrast, we would expect the observed soil thermal patterns to remain consistent across a range of soil and forest types.

### 3.2 Drivers of post-clearcut soil *CO*_2_ surface efflux and profile production patterns

While soil thermal conditions clearly drive overall soil *CO*_2_ efflux (Table 3) in the soils of these sites, an exponential relationship did not improve the strength of this relationship. This was unexpected as most studies observe stronger temperature-surface *CO*_2_ efflux relationships using an exponential model. Although previous studies within the region have also documented exponential relationships between these variables [40, 42], it has also been observed that seasonally averaged data provide a much stronger relationship than weekly data for soils of this region [58], an analysis that could not be carried out in this study with a 1 year dataset. The analysis of the layered mineral soil *CO*_2_ production was best described using exponential relationships through the upper part of the soil profile and linear relationships at depth, partcicularly as soil moisture increased (Table 4). However, the deep mineral soil contributions only represent a small fraction of the total (Fig 7b) and are unlikely to play a measurable role in driving the net soil *CO*_2_ efflux responses observed here.

The *CO*_2_ released as surface efflux attributed to the mineral soil component of respiration from each site arose from different processes. The clearcut sites were free of living vegetation and therefore represent a release of C from decomposition of SOM alone. In contrast, the paired forest sites represented a more complex set of C exchange dynamics, with both microbial and roots-related processes contributing to observed *CO*_2_ patterns [34–36, 39]. An order of magnitude estimate was made to remove the root signal from the soil profile by assuming 50% of soil *CO*_2_ efflux was generated by root-associated processes [34]. An exploratory study conducted at these sites in a separate experiment [59] suggests our estimate may have overestimated microbial contributions to total soil *CO*_2_ efflux by 10—20% during the growing season. This study also suggested that both microbes and roots responded positively to temperature to 15°*C* after which root responses did not increase in response to temperature. Therefore, the results presented in this study likely represent a conservative estimate of the differences between paired sites; it is possible that the clearcut sites may in fact be releasing an even greater proportion of *CO*_2_ from SOM decomposition relative to forest sites than we report here. While we are able to explore links between SOM decomposition and climate drivers, this dataset does not allow us to comment upon changes in substrate source and relative stability against decomposition across these study sites. Current debates in the literature surrounding the stability of SOM [13–16] certainly identify this as an important factor to consider in examination of these processes. Furthermore, while this study has focussed upon the immediate post-clearcut period, evidence suggests there may be cumulative effects of this landuse change within the soil profile SOM stores that are appartent several decades following clearcut harvesting in temperate forests of north eastern North America [17–20], particularly within the organo-mineral SOM fraction [21].

### 3.3 How does clearcut harvesting alter layered mineral soil *CO*_2_ production-climate relationships?

As soils warm, the standard relationship describing soil *CO*_2_ flux and temperature suggests rates of SOM decomposition should increase exponentially with soil warming. Further, theoretical relationships dictate an intrinsic temperature sensitivity of SOM [23] that should disproportionately affect pools of SOM housing more complex organic substrates [28]. Given that a greater proportion of these compounds are found at depth in soil profiles, we would expect these SOM stores to be most susceptible to changes in the soil thermal environment following clearcut harvesting. However, we also know that soil moisture can alter these theoretical responses to soil warming, producing an ‘apparent’ temperature sensitivity [23] in situations where decomposition becomes limited by microbial access to oxygen and/or substrates (e.g. [26]).

The soil heat *CO*_2_ production relationships from the 3-layered mineral soil profile production calculation demonstrate that subsurface processes are complex and generate responses to climatic controls that can be generalized based upon soil depth. Examination of the relationships for all forested and clearcut data, as well as site-specific data, illustrates the reduced sensitivity to heat through depth in the soil profile, a pattern that is enhanced at the clearcut sites (Table 4). This suggests that from a process perspective, an increasing dominance of the role of moisture in determining *CO*_2_ production that overwhelms the response to increases in soil heat.

The increased importance of soil moisture through soil depth in determining how decomposition will respond to increases in soil heat provides a mechanism that may provide some protection for deeper C in managed soils following clearcut harvesting. This was evident through the more persistent release of C in deeper mineral soil layers of the forested sites relative to their clearcut pairs (Fig 7), despite the fact that even at 1 m depth, clearcut soil temperatures could exceed those of their forest pairs by up to 3°C (Table 1; Fig 1). In drier regions, deep mineral SOM stores may be more susceptible to changes in the soil thermal regime following harvesting if soil moisture does not offer a similar level of protection against microbial decomposition.

It is unlikely that soil thermal conditions alone dicate SOM decomposition rates through depth in these mineral soils. In addition to alterations to the thermal environment within soils, clearcut harvesting also leads to other changes in the soil physico-chemical environment that may destabilize SOM, particularly in the mineral soil, where SOM is primarily associated with mineral phases. While priming effects arising from the transport of labile substrates and nutrients to depth in the soil profile following clearcut harvesting may play a role [37, 60], altered moisture conditions may also be implicated if they are shown to effectively reverse the podzolization processes under low redox conditions. In all likelihood the processes driving observed changes in SOM following harvesting are a consequence of multiple factors whose relative importance may shift in the years following harvesting. Even if SOM destabilization mechanisms are identified using controlled experiments, extrapolation back to a realistic field measure can be problematic(e.g. [61]) and must be constrained by field measurements.

## Conclusions

Examinations of soil organic matter decomposition processes in managed temperate forest soil profiles that are conducted *in situ* and consider the variability in the controlling relationships through depth are largely undocumented in the literature. This is due, in part, to the enormous technical challenges presented in carrying out such studies, requiring the collection of depth-intensive measurements which come at the expense of documenting spatial patterns. Here we observed an increase in soil temperature and moisture along with *CO*_2_ dynamics at four sites representing two forest-clearcut pairs within a representative humid temperate forest system located within the Acadian Forest Region of Atlantic Canada. While we observed a high degree of variability in *CO*_2_ concentrations, profile patterns were consistent with depth, and we were able to examine relationships between these *CO*_2_ dynamics, and the soil climate. Our analysis demonstrated that while heat was a primary driver of *CO*_2_ production, it became less important through depth and immediately following clearcut harvesting. A significant heat-*CO*_2_ production relationship could be only be established for a subset of depth intervals and sites using an exponential model in some cases (mainly within the upper soil), or a linear model in other cases (generally within the deeper mineral soil). The breakdown in the exponential heat-*CO*_2_ production relationship was systematic with depth, and observed to be a function of increased soil moisture. These findings have implications for how we model soil SOM dynamics, and predict changes in SOM stability within soils, particularly harvested deep mineral soils.
